# Childhood Injuries in Pakistan: Results from Two Communities

**DOI:** 10.3329/jhpn.v28i4.6046

**Published:** 2010-08

**Authors:** Seema Lasi, Ghazala Rafique, Habib Peermohamed

**Affiliations:** ^1^ Human Development Programme, Aga Khan University, Karachi, Pakistan; ^2^ Human Development Programme, Community Health Sciences, Aga Khan University, Karachi, Pakistan; ^3^ Human Development Programme, Internal Audit Department, Aga Khan University, Karachi, Pakistan

**Keywords:** Child, Cross-sectional studies, Injuries, Retrospective studies, Pakistan

## Abstract

The study aimed at determining the incidence, nature, and extent of childhood injuries in two suburban and rural communities of Pakistan. The findings of the study are based on a cross-sectional survey of 2,292 children aged 1-8 years. Information was sought retrospectively from the primary caregiver on the occurrence of injury that required formal or informal medical consultation during the past three months. The incidence rate of non-fatal injuries that required care outside home for children aged 1-8 years was 19.7 injuries per 100 person (child)-years of exposure [95% confidence interval (CI) 16.41-23.51]: 26.5 injuries per 100 person (child)-years of exposure (95% CI 21.31-32.63) in the suburban area and 12.1 injuries per 100 person (child)-years of exposure (95% CI 8.68-16.66) in the rural area. The most common non-fatal injuries were falls (10.5 fall injuries per 100 person (child)-years of exposure), burns and scalds (3.5 burn injuries per 100 person (child)-years of exposure), and road traffic injuries (RTIs) (2 RTIs per 100 person (child)-years of exposure). One fatality due to drowning was also reported during the study period. The difference among sex was highly significant (p=0.03). Boys (60%) were at a higher risk of injuries compared to girls (40%). The data also revealed that 61% of the injuries took place inside the home. The magnitude of childhood injuries in the two communities was significant compared to the findings of the National Health Survey of Pakistan (1990–1994). The fact that the majority (61%) of the injuries occurred inside the home raises many questions in relation to the household hazards and adequacy of safety and child-proofing measures in these households. There is a need to develop community-based interventions, creating awareness about the consequences of childhood injuries and educating families about preventive measures to reduce the incidence of injuries during early and middle childhood.

## INTRODUCTION

Injuries are a leading cause of death and disability (1–3), with more than five million deaths each year (4). It is a significant public-health problem in terms of morbidity, mortality, and life-long disability.

Children are most vulnerable for injuries and violence. (According to the Convention for Rights of Children, “a child means every human being below 18 years of age”). In 2004, the estimated annual mortality specific to injuries and violence alone was 950,000 (5) (The author of the report has asserted that the figures are highly underestimated). More than 95% of these deaths occur in low- and middle-income countries (LMICs) (6), accounting for 40% of all childhood deaths (5).

Non-fatal childhood injuries impose a significant burden on morbidity worldwide; 400 million children are estimated to suffer from non-fatal injuries every year (7). Disability-adjusted life-years (DALYs) are lost due to road traffic injuries, and falls rank among the top 15 causes of the global burden of diseases (5). Non-fatal injuries may also lead to brain damage and permanent disability, which has life-long consequences. Injuries also have a very strong negative impact on children's development during the early years (8).

According to a report by the UNICEF Innocenti Research Centre, in Asia alone, the estimated rate of mortality of children, aged up to 18 years, due to injuries exceeds 1,000 per 100,000 people (9). The report further elaborated injury as the second leading cause of mortality of children aged less than five years and the major leading cause for children aged 1-5 years (9).

Pakistan is identified as a high-risk country of injury-related mortality for children and adolescents, with an estimated mortality rate of 30+ per 100,000 people (6). According to the National Health Survey of Pakistan 1990-1994, the incidence rate for non-fatal injuries among children aged less than five years is 49 per 1,000 children per year (10).

Data on childhood injuries have been reported by a few studies from Karachi, a cosmopolitan city of Pakistan, with over 16 million people. One study identified 1,320 cases of injury (≤15 years old) which required emergency medical transportation during 27 months; 15% of these cases died before reaching hospital (11). This information was collected from the ambulance service log book. Nevertheless, there is a high probability of under-reporting based on service-delivery as it cannot be expected to capture injury-related events in its entirety compared to the information from community-based studies (12,13). Another community-based study in squatter settlements reported a non-fatal injury rate of 37 per 1,000 persons per two months for children aged less than 15 years (14).

The study on childhood injuries in suburban areas and rural communities of Pakistan was designed to report community-based incidence of morbidity due to injuries, exclusively for children aged 1-8 years for one suburban community in Sindh and a rural community in Balochistan over a three-month period to draw some inferences about the magnitude and types of major childhood injuries among early and middle childhood (1-8 years).

The study is particularly useful as recent information on the magnitude, nature, and causes of childhood injuries from rural and suburban communities of Pakistan is not available. This study will, therefore, enable a better understanding of childhood injuries in rural and suburban communities and would help develop evidence-based interventions directed towards preventive measures needed to successfully reduce such occurrence. This paper provides information on the incidence of non-fatal childhood injuries, the nature and the extent of injuries, identification of sources inside and outside the house causing these injuries, and the healthcare-seeking behaviour of caregivers.

## MATERIALS AND METHODS

The study was undertaken in Tandojam district, Sindh province and in Mastung district, Balochistan province. Tandojam is about 20 km from the main city of Hyderabad, with most of its population being associated with agriculture. Mastung is about 100 km from the main city of Quetta, the provincial capital of Balochistan and has a mountainous terrain with no availability of basic amenities. The literacy rate of Tandojam is 65% compared to 41% in Mastung.

The Aga Khan University–Human Development Programme (AKU-HDP) is running a community-based early childhood development (ECD) programme in these two districts since 2005. The programme regularly monitors the growth and development and provides advice to primary caregivers for a cohort of 2,865 children aged 0-8 years. The present information is based on a cross-sectional survey conducted in September 2007.

The survey was conducted on the entire sampling frame of 2,865 children enrolled in the ECD programme with the AKU-HDP. The respondents were the children's primary caregivers, and information was collected retrospectively based on any injury that had happened to the child during the last three months (11), which required either formal or informal medical consultation outside the home (13). Surveys on injuries recommend the three-month duration for collecting information to minimize recall bias (15). Information was collected using a structured questionnaire, specifically developed for this purpose. The questionnaire was translated into local language and then back-translated into English and pretested to assess and evaluate its effectiveness before administration in the field. Informed consent was taken from caregivers with assurance of complete confidentiality.

The total number of children, aged up to eight years, who were considered for data collection, was 2,865. Data pertaining to children aged ≤1 year were, however, excluded from analysis as the nature of injuries affecting this category of children was very different. Thus, the net number of children, aged 1-8 years, who were approached for collection of data, was 2,355. Of the 2,355 caregivers of children, 97% consented to participate in the study. The present analysis was based on a total sample-size of 2,292 children. Although the caregivers were specifically asked about those injuries only which required professional care, many also reported injuries for which care was not sought. All responses provided by the caregivers were noted; however, those injuries which did not require professional care outside the home were excluded during analysis.

Data were collected by the early childhood development workers (ECDWs) who are already engaged in home-visits in the ECD programme. One-day training was provided to these ECDWs to develop an understanding of the issue and for the comprehension of the questionnaire. The field supervisors and the data-management personnel supervised data collection.

The main focus of the study was childhood injuries, irrespective of whether these were intentional or unintentional. Mixed instances are, therefore, reported here without discernment of the type.

For quality assurance of data, consistency checks on completeness and accuracy of collected data were ensured by the field supervisors and data-management staff.

Data were double-entered using the Visual FoxPro software (version 6.0) and analyzed using the SPSS software (version 13 and 16). To study the characteristics of the subjects, descriptive statistics were generated for the entire range of variables under study. Most variables were categorical for which proportions were reported, and means and standard deviations were calculated for continuous variables.

## RESULTS

The demographic characteristics of the study participants are presented in Table 1.

**Table 1.** Demographic characteristics of children aged 1-8 years (n=2,292) in the suburban community of Sindh and the rural community of Balochistan

Characteristics	Frequency (n=2,292)	%
Area of residence		
Rural	1,085	47.3
Suburban	1,207	52.7
Sex		
Male	1,176	51.3
Female	1,116	48.7
Age (years)		
1-2	348	15.2
>2-3	342	14.9
>3-4	343	15.0
>4-5	362	15.8
>5-6	357	15.6
>6-7	342	14.9
>7-8	198	8.6

During the study period, 113 episodes of injuries required professional care outside the home; one case was a fatal injury. Thus, the incidence rate for non-fatal injuries for children aged 1-8 years was 19.7 per 100 person-years [95% confidence interval (CI) 16.41-23.51]: 26.5 per 100 person-years (95% CI 21.31-32.63) in the suburban area and 12.1 per 100 person-years (95% CI 8.68-16.66) in the rural area.

The caregivers reported 106 episodes of injuries for which they did not seek any outside care. The majority of these injuries included falls (n=54), hit by something (n=11), road-traffic injuries (n=10), cuts from sharp tools or objects (n=15), and more.

**Fig. 1.**
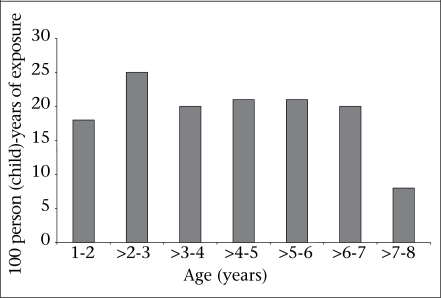
Distribution of non-fatal childhood injuries according to age in suburban and rural communities of Pakistan (n=2,292)

Injuries in the suburban areas were more than double compared to injuries in the rural area (p<0.001). More boys encountered injuries (60%) than girls (40%), and the difference was significant (p=0.03). Figure 1 shows the distribution of the injuries according to age of the study participants. The figure also shows that, during the second year, the children had the highest number of injuries; however, the difference was not significant.

The most commonly-occurring non-fatal injuries were falls (10.5 fall injuries per 100 person (child)-years of exposure), burns and scalds (3.5 burn injuries per 100 person (child)-years of exposure), and road-traffic injuries (2 RTIs per 100 person (child)-years of exposure). Falls also were the most predominant cause of childhood injury for all ages. This includes falling from bed (23%), falling from baby-cot (8%), falling on floor (15%), and falling from staircase (12%). Moreover, of the 20 non-fatal injury events, 60% of burns and scalds were due to boiled liquids, and the remaining injuries occurred due to fire. Road-traffic injuries were mainly due to motorbikes (36%), bicycle (27%), car (18%)), autorickshaw (9%), and pedestrians (9%).

**Fig. 2.**
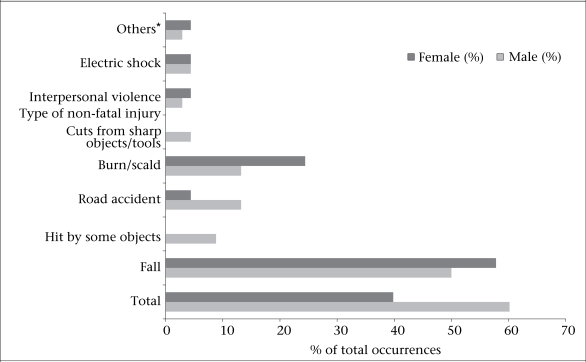
Distribution of non-fatal childhood injuries according to sex among children aged 1-8 years in the suburban and rural communities of Pakistan (n=2,292)

Figure 2 depicts the distribution of injuries according to gender. For overall injuries, boys dominated girls. However, for certain injuries, such as burns and scalds, girls had twice as much injuries compared to boys. Burns and scald injuries for which the degree of burns could not be ascertained, 50% occurred in the kitchen. A child was also hospitalized for 10 days for burn injuries and was still under treatment when the information was collected. No girl child encountered injuries that was caused due to getting 'hit by something’ or cuts from any ‘sharp tools or objects’. Although a difference among sex was observed for certain categories, it was not statistically significant probably due to a small number of occurrences. The data also revealed that boys encountered three times more road-traffic injuries than girls. Moreover, 81% of the road-traffic injuries occurred in the suburban area. The category ‘others’ in figure 2 includes injuries due to poisoning, animal-bite, and close to drowning.

*Others include: poisoning, animal-bite, and close to drowning

### Characteristics pertaining to cause of non-fatal injury

Table 2 provides detailed characteristics of injuries. The table shows that about 61% of the injuries occurred inside the home, with 31% occurring in the courtyard, 11% on the staircase, and 11% in the kitchen. The major location of injuries outside the home was roads or streets (26%). Irrespective of the place of occurrence, in 67% of the injury cases, the child was either alone or with peers but without any supervision by an adult.

**Table 2.** Characteristics of non-fatal childhood injuries among children aged 1-8 years in the suburban community of Sindh and the rural community of Balochistan (n=2,292)

Characteristics	Frequency (n=113)	%
Place of injury		
In the home	69	61.1
Outside the home	44	38.9
Area of occurrence of injury		
Courtyard	35	31.0
Stairs	12	10.6
Kitchen	13	11.5
Bedroom	8	7.1
Balcony	4	3.5
Playground/park	6	5.3
Road/street	29	25.7
School	3	2.7
Others*	3	2.7
Adult accompanying at the time of injury		
Yes	37	32.7
No (alone or with person aged ≤15 years)	76	67.3
Care-seeking behaviour		
Rural Health Centre/Basic Health Unit	12	10.6
Hospital	14	12.4
Private doctors	85	75.2
Traditional healers/ paraprofessionals	2	1.8
Duration (days) of recovery		
Mean±SD	15.69±13.24	
Still under treatment	16	14.15
Hospitalization due to injury		
Yes	2	1.8
No	111	98.2
Absenteeism from school (n=43)		
Number of children absent due to injuries	23	53.5
Mean days±SD of school absenteeism	9±10	
Median days of school absenteeism	7	

SD=Standard deviation

Results of analysis of healthcare-seeking behaviour after the occurrence of injury showed that the large majority (75%) of these children were taken to private doctors whereas the remaining children were either taken to the government health facilities, traditional healers, or paraprofessionals. A marked difference was observed in using the available government health facilities. Not a single case of injury was taken to the Basic Health Unit (BHU) in the rural areas. The rural community preferred the hospital, which is 12 km away, over the BHU. On the contrary, the suburban communities preferred the Rural Health Centre (RHC) over the tertiary-care hospital, which is 40 km away from the area. Furthermore, analysis of healthcare-seeking behaviour for the suburban and rural communities showed a 30% higher use of private doctors. The mean±standard deviation (SD) number of days for recovery from injury was 15.69±13.24 days whereas 16 children were still under treatment at the time of data collection. Two children were also hospitalized following the injury, and the maximum duration of stay in hospital was 10 days. Based on the information given by the caregivers, 43 children were enrolled in schools. Of these, 23 (53.4%) were reported for school absenteeism, and the mean±SD number of days of school absenteeism was 9±10. The total number of school days lost due to injuries was 217 person-days.

## DISCUSSION

The study has provided the incidence rate of non-fatal childhood injuries in the selected suburban and rural communities of Sindh and Balochistan. It has also provided an understanding of the nature and practices of caregivers. These findings will enable the development of interventions targeting the reduction in the number of childhood injuries.

The incidence rate of non-fatal injuries for children of both the communities aged 1-8 years cannot be compared with any other studies in Pakistan due to the non-availability of data for the specific age-group. However, compared to the National Health Survey of Pakistan (NHSP) 1990-1994—analysis of children aged 0-5 years—the study data report a four-time higher incidence rate (10). There could be several considerations for this. First, the findings of the NHSP on childhood injuries are more than a decade old. Second, the recall period for the NHSP was one year compared to three months in the present study; the three-month period is most desirable and highly recommended to minimize recall bias (16).

Further, the higher risk for injury-related incidence, particularly for 0-15 years old children, was also highlighted by the population-based National Injury Survey of Pakistan (NISP) 1997 (15); the child and adolescent age-group ranks the second major high-risk group for non-fatal injuries in the country. However, the NISP does not provide a break-up of injuries for children and adolescence. Another community-based survey from Karachi reported the incidence of major injuries for the age-group of ≤15 years to be 37 per 1,000 injuries for a recall period of two months (14), which is very much parallel to the results of our data.

The results of the present study also showed similar trends to other community-based studies outside Pakistan. The results of a community-based study on children aged 1-5 years in Damascus, Syria, reported the incidence rate of 230 per 1,000 persons per year (for both types of injuries, requiring medical treatment and those treated in the home) (17).

Of the most commonly-occurring non-fatal childhood injuries, ‘fall’ was the leading cause, followed by burns and scalds and road-traffic injuries. Similar findings were reported by the UNICEF Innocenti Research Centre in its report on injury incidence in six countries of Asia (7).

The occurrence of burn injuries among girl children more than twice, with half of them occurring in kitchen, raises several questions about the cooking and related work these girls are doing at this tender age or caregiver's behaviours regarding young children, especially girls, and also the measures adopted by their caregivers for their safety, which certainly needs further investigation. Similarly, the high number of road-traffic injuries among boys requires further exploration to determine the causes. One of the reasons might be the non-availability of safe play-areas and parks, because of which these children are forced to play on streets and roads, and consequently, many encounter road injuries. Furthermore, the high occurrences of road-traffic injuries in the suburban areas, as these areas are closer to highways, also raises serious concerns about the effectiveness of road-traffic safety measures in these areas.

The data did not reflect significant differences in the nature of injuries for the two geographical locations, except for the incidence rate of non-fatal injuries which were more than double and a high frequency of road-traffic crashes in the suburban areas. Based on this, it would be interesting to assess and ascertain the current trend of childhood injuries in the urban areas of Pakistan. The geographical differences were also observed in a study from Uganda which reported a high incidence of injuries in urban areas (18).

Keeping in mind that a child spends a significant amount of time inside the home and that the home is considered a safe heaven for the child, the findings of this study simply raise many questions in relation to the adequacy of safety measures inside the home. The study clearly indicates the places where the majority of injuries occurred were inside the home, which is an indication of prevailing household hazards and inadequate safety measures of child-proofing in these households. A study in Iran also reported an excess of home-related injuries for children aged 0-4 years (19).

The findings of the present study also underscore the need for full-time supervision by an adult, especially during this vulnerable age. A study in Jordan identified the presence of an adult to be a significant factor to reduce the occurrence of injuries, especially in early and middle childhood (20). Supervision by an adult is an important factor but the safe environment cannot be ignored as a key factor in reducing the number of childhood injuries.

### Limitations

The study had a couple of limitations. Most injuries may not be physical in nature or considered to be minor by the caregiver, and as such, medical care might not have been sought after the occurrence of injuries. Therefore, the incidence, extent, and nature reported by the caregivers could be an underestimation. Another possible cause of underestimation could be the accessibility of healthcare provision in terms of financial, social and cultural barriers, especially for mothers and children. Keeping in view that a large number of injuries for which no outside care was sought, it is critical to mention a realization that care-seeking behaviour could not only be a function of the severity of injury but also largely depends on accessibility and affordability on part of caregivers of the injured child. This insight can be further explored in future studies.

Information on the incidence of injuries was collected retrospectively which might have caused an under-reporting due to recall bias; however, this limitation is inherent in all community-based surveys (13).

### Conclusions

Compared to the results of the NHSP 1990-1994, the high magnitude of non-fatal childhood injuries reported in this study, together with the possibility of underestimation, suggest that childhood injury is a far more significant problem than reported by earlier studies, and there is a dire need to look into ways to overcome this growing predicament. Non-fatal childhood injuries have a significant magnitude in both the communities and need to be addressed urgently.

An active surveillance system to monitor injuries is highly recommended to obtain a further understanding of childhood injuries and to guide the development of interventions accordingly. An important area for future research would be to assess hazards in and out of the home, which are the potential causes of injury among children. Viewing the current magnitude of childhood injury, it is imperative to develop and assess the effectiveness of community-based injury interventions to reduce its incidence. An important component of this intervention should be to educate families about the consequences of injuries and the preventive measures to mitigate the risk. The interventions should be directed towards parental education for behavioural change and modifications of the home environment.

## ACKNOWLEDGEMENTS

The authors acknowledge the funding provided by the Royal Netherlands Embassy for the Releasing Confidence and Creativity (RCC) project under the umbrella of the Aga Khan Foundation, Pakistan. The same funding source was used for carrying out this study. The authors are thankful to Mr. Iqbal Azam, Assistant Professor of Statistics, Aga Khan University, for his inputs in statistical analysis and Dr. Sheridan Bartlett for her review of the article and her valuable comments for its improvement.
